# Playing the harmonica with chronic obstructive pulmonary disease. A qualitative study

**DOI:** 10.1177/14799731221083315

**Published:** 2022-04-12

**Authors:** Adam Lewis, Joy Conway, Jack Middleton, Chris K Startup, James Wyatt

**Affiliations:** 1 Department of Health Sciences, 3890 Brunel University London, Kingston Lane, UK; 2 Faculty of Health, Education and Society, Waterside Campus, University Drive, 6087 University of Northampton, UK

**Keywords:** COPD, qualitative, harmonica, arts in health

## Abstract

**Objectives:** To investigate the experience of playing the harmonica for individuals with COPD.

**Methods:** A qualitative, phenomenological study using semi-structured interviews and reflexive thematic analysis.

**Results:** Eight people living with COPD (six females, two males) were recruited, who had attended at least six weeks of harmonica group sessions, either face-to-face prior to the COVID-19 pandemic or remotely. Five themes were generated. Themes included ‘hard in the beginning’, ‘holding the condition’, ‘breathing control’, ‘gives you a high’ and ‘needing the Zoom class’.

**Discussion:** Playing the harmonica with COPD is difficult at first, particularly drawing a breath through the harmonica. With practice, experience in a fun activity and quality teaching, individuals were able to become more attuned and embodied with their breathing, and playing the harmonica offered a breathing control strategy. Songs, rather than breathing, became the focus, and participants were able to escape living with respiratory disease when playing. Participants reported the harmonica helped mucous expectoration. The group was a priority in the weekly lives of participants, even though the ‘buzz’ of being part of a group was lost when participating online. Further mechanistic studies and randomised controlled trials are needed to investigate the biopsychosocial benefits of playing the harmonica with COPD.

## Introduction

Chronic Obstructive Pulmonary Disease (COPD) is one of the largest causes of morbidity and mortality globally.^1^ Evidence-based interventions are available to help treat and manage disease burden, such as flu and pneumonia vaccinations, smoking cessation, inhaled therapies, pulmonary rehabilitation (PR) and lung volume reduction.^2–4^ However, patient adherence to COPD therapies which require significant patient engagement, including smoking cessation, using inhaled medication, and participating in pulmonary rehabilitation, is suboptimal.^5–8^

Participatory arts-in-health interventions such as singing, dancing and theatre (performing arts) are recognised as being beneficial for health outcomes by the World Health Organisation.^9^ Singing for Lung Health (SLH) is an arts-in-health intervention with evidence for improving physical health in respiratory disease.^10^ Other arts-in-health interventions such as dancing and other music therapies maybe appropriate intervention choices for individuals with chronic lung diseases.^11–13^ The harmonica is a hand-held instrument requiring the player to draw and blow against resistance in order to produce a tune. To draw breath refers to the action of inhaling while the mouth is sealed over the harmonica and blowing refers to the action of exhaling into the harmonica. When harmonica playing has been trialled in combination with PR compared to participating in PR only, no statistically significant differences in respiratory muscle pressures, exercise capacity or quality of life were observed.^14^ The authors stated that the lack of statistical differences seen in these outcomes may be due to not having played the harmonica with sufficient dosing and the small sample size. Inspiratory muscle training effects are also expected from PR.^15^ Hart et al.^16^ performed a cohort study with individuals with COPD who participated in 12 weeks of harmonica sessions. These individuals gained clinically and statistically significant improvements in their respiratory muscle strength (PImax mean difference = 15.4 cm H20 (P: 0.0017), PEmax mean difference = 14.4 cm H20 (P: 0.0061))^17^ and walking distance (6 min walk test mean difference = 61 m (P: 0.03)),^18^ but there was no control group for comparison. The limited quantitative evidence available suggests that playing the harmonica has potential clinical value as a participatory arts intervention for people living with COPD. The intervention may also increase intrinsic motivation to adhere to beneficial self-management activity as an enjoyable hobby. However, experiences of playing the harmonica with COPD are not known. The purpose of this study was to investigate the experience of playing the harmonica for individuals who are part of a harmonica group specifically created for people living with chronic respiratory disease. Experiences of being part of a group included both face-to-face participation before COVID-19 and the change to remote group delivery because of the pandemic.

## Methods

### Design

This was a qualitative study using an interpretivist, phenomenological stance. The phenomenology was influenced by Merleau-Ponty regarding corporeality, with the body as a perceiver and actor^19^ and also by Van Manen^20^ with a view to practice, with the intent of caring for individuals with respiratory disease.

### Participant recruitment

Individuals were recruited from a harmonica group in the UK, consisting of individuals living with different chronic respiratory diseases. Participant information sheets were given to all individuals in the group diagnosed with COPD who had participated in at least six weekly sessions. Interested individuals provided informed consent. AL had no previous relationship with recruited participants. Ethical approval for the project was obtained by Brunel University London College of Health, Medicine and Life Sciences Research Ethics Committee (25,578-MHR-Dec/2020–29,450–2). Out of a regular harmonica group attendance of 30 individuals, 15 had known COPD and were approached to enter the study. Of those recruited, five participants reported previous participation in PR or maintenance exercise programmes, and all participants reported participation in SLH groups.

### Data collection and analysis

Semi-structured interviews were performed by AL via Zoom or telephone depending on participant preference. The semi-structured interview guide is provided in the online supplement. Interviews were performed between January and April 2021. Interviews were transcribed verbatim into Microsoft Word, and reflexive inductive thematic analysis was performed using Microsoft Word and Excel, including the stages of data familiarisation, coding, generating initial themes, reviewing and defining themes and writing the report.^21–23^ Respondent validation or peer checking of themes was not performed because this was deemed not appropriate for the interpretivist nature of the research. The harmonica group leader assisted in recruitment but was not involved in the analysis of the interviews.

### Setting and context

Groups were set up by an occupational therapist (JW) and a musician (CS) who have previously been trained to run groups specifically for individuals with chronic respiratory disease (CRD) by the British Lung Foundation. All weekly harmonica sessions consisted of warm-up exercises and performing different songs specifically chosen by the harmonica group leader to be appropriate for individuals who may become breathless. Harmonica sessions lasted 1 hour per week. All harmonica sessions were run via Zoom at the time of recruitment because of COVID-19 restrictions.

## Results

**Figure 1.**
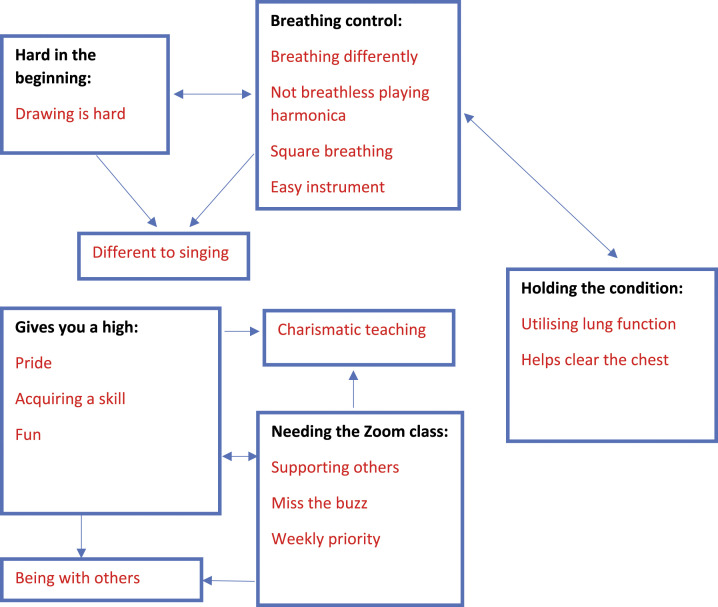
Thematic map.

Eight participants (six females, two males) were recruited. Interviews lasted an average of 49 min (Range: 40–64 min). Five themes were generated from thematic analysis which included ‘Hard in the beginning’, ‘Holding the condition’, ‘Breathing control’, ‘Gives you a high’ and ‘Needing the Zoom class’. Each theme will be presented below with example quotes and analysis. The themes in bold text and subthemes in red text are presented in the thematic map (Figure 1). Further example quotes are provided in the online supplement.

### Hard in the beginning

Participants reported that altering their breathing in combination with trying to hit the right note was difficult. Many found that they were putting too much effort in to make the sound.

‘first you find it hard but then once you get to realise how to breathe properly you’re actually playing a song without realising and you are not feeling breathless afterwards’

P1, page 3, line 53, played harmonica since 2018.

There is a skill playing the Harmonica properly, and the skill can be judged by the ability to play a tune, but also in combination with the ability to not get too breathless.

‘When I first started and found it so hard, I couldn’t breathe to get enough breath to blow the harmonica, until I was shown how to do it’.

P2, page 3, line 8, played harmonica since 2018.

This suggests that playing the harmonica correctly is not intuitive for individuals with breathing difficulties and it requires a particular way of breathing to activate the musical notes. The main difficulty was drawing the breath on the inhale to overcome the resistance of the harmonica:

‘Blowing is okay, it's drawing, and if you've got to do it very quickly, you can't really take a good breath you know what I mean, when you're breathing, in an out, it is much harder to do it through the harmonica’.

P6 page 3 line 58, played the harmonica for 2 months.

When breathing in against the resistance of the harmonica (drawing the breath), it disrupts the normal un-phonated breathing pattern. This is a challenge for individuals with COPD.

‘because I'm drawing too much in at once, or I'm not breathing out enough because you’ve got to breathe out to draw in’.

P8 page 4 line 86, played the harmonica for 2 months.

### Holding the condition

Participants reported that they felt playing the harmonica regularly was a way that they were able to give their lungs a work-out and hold the condition, preventing them from worsening.

‘I think it’s the structure that he puts in it, and he tells us you know how to do it, and to breathe, and to get the best out of our breathing. But I honestly don't think I would be in the position I am now if I was still coughing and only on the medication that I was on, I think I would be in pretty dire straits’.

P6 page 8 line 174, played the harmonica for 2 months.

Playing the harmonica can act as a therapeutic intervention with perceived health benefits. Playing the harmonica was thought to be a way of preventing symptoms worsening.

‘It’s making me exercise when perhaps I wouldn't do those exercises, because you know when I take my ventolin in the morning, because of harmonica, I now do their exercises for breathing and that helps me take my ventolin in my inhaler in the morning…Because you’ve gotta build up your lungs and capacity because you’ve lost you know, you’re losing it, so I want to keep it and I can’t get what i’ve lost, that, but I want to keep it at the level how I am at the moment. I don't wanna have to go onto oxygen like some of the ladies have, so I’m fighting it’.

P8 page 9 line 211, played the harmonica for 2 months.

This individual commented how playing the harmonica is a way of potentially preventing lung function decline and improving inhaler technique. The harmonica could be used as a training aid. This is understandable because both pressurised metred dose inhalers and the harmonica require coordination of the device and sufficient inhalation effort. Participants reported that playing the harmonica helped to clear mucous off their chest which helped in holding the condition on a day-to-day basis. The participant below compares using the harmonica with other aids supporting the management of their COPD, including an inspiratory muscle training device and a positive expiratory pressure (PEP) device. They preferred playing the harmonica compared to other devices:

‘forget I've got any chest problems when I'm playing the harmonica. I find the trainers and the flutter not as easy for me, I'm on the very, the thing with the spring, I'm on the very low level, I can't seem to get up, so it's strange that, put the harmonica into my lips and I'm fine, yes I do get out of breath (if) I played a lot, but that's to myself. Because I play different things to the class but it's not, I feel like it's not a chore, the flutter and this the other one, I find I don't do them regularly. I probably don't need to because of using that harmonica’.

P4 page 8 line 184, played the harmonica since 2017.

### Breathing control

A sense of needing to control the breath to play the harmonica or gaining a sense of controlled breathing through playing the harmonica was a central theme in this study. Playing the harmonica was thought of as breathing differently, and often a way to forget about being breathless at all.

‘it's a lot lighter, it's like I said before, it's slightly whispering. It's like you whispering in, it's like you're actually whispering into your harmonica so it's not as hard….when you realise it's coming, just coming from your throat area, and not down here, you tend to just blow into it gently’.

P1 page 5 line 110, played the harmonica since 2018.

Breathing ‘from your throat’ and gently appears to contrast with the commonly used ‘diaphragmatic breathing’ exercise technique more commonly to individuals with COPD.

‘I forget that I have breathing problems when I do it, when I play, that’s what it means to me’.

P3 page 14 line 321, played the harmonica since 2017.

Playing the harmonica is a way of living without respiratory disease and a temporary respite from symptoms for some.

Through music, individuals are becoming more embodied with their breathing in a task where the product of action is not their breath, but the tune. This tune has no disease attributed to it and so participants lose focus on their own disease.

‘It's making your head work use your lungs, and I think that's what we tend to miss out, that connection, when you panic, and you can't breathe, and you panic and that's it. So there's no control up here at all, it is just one overriding panic, so doing anything that can help you ease that control and use that control, it’s got to be a fantastic thing for anybody with their respiratory problem’.

P5 page 19 line 441, played the harmonica since 2017.

Playing the harmonica shifts the cognitive focus on using the breath constructively as a tool to enable the sound to be heard. There appears to be a degree of breathing control which can be achieved and mastered, and breathing control appeared to be determined by the ability to concentrate on playing the right notes.

‘You've got to get your breath right in it, when you're following the notes. Because it's so easy to go off to get the wrong note. So concentration is very, is very, is another element… I'm going straight in on the notes not worrying about the breathing, I'm trying to get a notes right’.

P3 page 18 line 387, played the harmonica since 2017.

Participants get feedback from the tune regarding the quality of their breathing:

‘if it's clear, if it's what I want it to be, and how I feel it should sound, and all how I know it sounds when you know a tune, you know exactly how it should sound. When I achieve that, then I know that my breathing is good’.

P5 page 10 line 227, played the harmonica since 2017.

This participant highlights the important association between the aesthetics of the performance of harmonica playing and the quality of breathing. If the performance sounds good, then the participant understood themselves to be breathing well.

With more experience playing the harmonica, individuals commented that breathing whilst playing the harmonica felt significantly different to breathing during exertion or singing.

‘I'm about 70% of normal, but you don't need an awful lot of power to play the harmonica from what I've picked up. I may be wrong, but you don't blow in really hard. Not like blowing the trumpet where you know, gotta have a lot of breath, so that's more so, than singing, where you've got to perhaps use a lot more of your body….Whereas, harmonica is, go, go for it’.

P4 page 12 line 279, played the harmonica since 2017.

‘When you're young you know you don't think about your breathing it just happens doesn't it, or when you can't do it, it’s quite frightening, you know it really is, you think, you know, what's happening here? Now, I now know how to control it a lot better, and how to get over these shortness of breath times, you know. And I think this has helped, the harmonica, because it pushes me, the singing as I say always sung, so it's not too much of an effort’.

P6 page 16 line 378, played the harmonica for 2 months.

Not everyone forgot about their breathing or found it easier when playing the harmonica. The participant below had not played the harmonica for years like some of the others:

‘I found the singing better really because although it might make me cough, I do that more, lungs are more open after the singing, whereas with the harmonica I feel like my chest is quite tight, so I feel the singing is doing my lungs more good really, and I feel I have a better result after singing lesson than the harmonica’.

P7 page 5 line 109, played the harmonica for 2 months.

The difference in experience of symptoms between singing and playing the harmonica could be due to differences in the breathing techniques used, but also the differences between the sound being made by one’s own body compared to an instrument.

### Gives you a ‘high’

Playing the harmonica is fun, it is enjoyable and it gives people a ‘buzz’. Players are left on a ‘high’, full of positivity. The ‘high’ feeling is a sense of being proud in achievement, gaining a new skill, having self-belief and being able to perform a tune in a world outside of disease, without anxiety.

‘What a buzz, there's nothing like it at all. You know we come away, we usually go to the pub afterwards because it’s nice, you come away because you're still on a high’.

P5 page 15 line 374, played the harmonica since 2017.

‘When I play that, you know, you feel so comfortable in yourself you actually feel comfortable in yourself because you sitting, that, it's like you're in a world of your own, and that’s basically what it is. We start to feel comfortable in your own body and relax and you are more relaxed in your own body you know. And you get more confident as well. The more that you play the more confident you get’.

P1 page 14 line 335, played the harmonica since 2018.

Playing the harmonica improves how people feel about themselves, which is related to being comfortable in their own bodies. Part of being on a ‘high’ is the escapism of being enthralled by the charismatic teacher.

‘(Name) makes it very entertaining and at the end of the day I can get a bit of a tune out of the harmonica, all be it not very good, but it's just it's just good fun, and I feel that because I'm struggling too with the in breaths I feel that it's maybe doing some good because I'm breathing out to breathe in through this, and it is, you have to make a bit more of an effort. I enjoy it, and that's the reason why you do something’.

P6 page 6 line 121, played the harmonica for 2 months.

Having a charismatic leader who makes sessions fun is a reason to continue attending the group. It is not surprising that individuals had been in the group for years.

### Needing the Zoom class

Participants discussed the necessity, advantages and disadvantages of playing the harmonica online. Being part of the online group became a priority in their weekly schedule.

‘You're in a group in a room it's more, it can be more embarrassing I suppose, if you go wrong, whereas when you're online he always says don't worry it's just me’.

P4 page 13 line 311, played the harmonica since 2017.

‘Harmonica is brilliant, and it's, let's keep the fact it's keeping us alive you know, with the hope that you know we're going to be together, we're going to be doing it again and will be physically together again’.

P3 page 8 line 178, played the harmonica since 2017.

The quote above illustrates the temporal and social relatedness of the experience of playing the harmonica online. It enables people to look to their future with positivity, at the same time reflecting positively on previous experiences of being physically together with others. The meaning of weekly group attendance transcends being in the moment of the session. However, others felt isolated by online participation and missed the genuine face-to-face group contact:

‘I miss that banter…it's just missed that, that impromptu chatter and just being with people I think makes an awful difference. It makes really good difference. But the Zoom is extremely good considering our circumstances you know. If we didn't have all this technology we would be in terrible states, I mean I would be here I wouldn't have spoken to anybody for nearly a year’.

P6 page 9 line 209, playing the harmonica for 2 months.

## Discussion

The following discussion further explores the thematic analysis in relation to the interpreted phenomenological and clinical meaning, and with reference to previous published literature. Discussion content has been structured within the meaning of being ‘Attuned to breathing’, ‘A social need’ and using the harmonica as ‘An adjunct’. We then discuss the strengths and limitations of the study.

### Attuned to breathing

This qualitative analysis explored the experiences of individuals playing the harmonica with COPD. No previous investigation of harmonica playing has been explored in individuals with COPD. The main theme from the analysis was that playing the harmonica both requires and provides a sense of breathing control. This is because it is necessary to breathe differently through the harmonica with concentration on connecting the breathing with producing sound. Music therapy has shown to be effective at improving breathlessness for individuals with COPD.^24–29^ However, there is very limited quantitative data and no qualitative data from harmonica trials to the authors’ knowledge. The melodica may have some similarities with the harmonica regarding breathing control. Okamoto et al.^30^ investigated the use of a melodica used to perform exhaled breathing exercises in addition to PR in a randomised 4-week crossover trial with the control group performing additional leisure time activities such as reading or watching television. Melodica playing was associated with improvements in peak expiratory flow (1.53 L/s to 2.47 L/s) and FEV_1_% (52.8–64.94). The authors claim that these improvements were possibly attributed to the fact that expiratory lung volume was easier to control with practice due to the sound feedback provided by the instrument. However, the sample size of this study was very small with data analysed from 13 out of 21 participants and therefore no generalisable conclusions can be drawn. Participants in our study felt that the altered breathing was helping to clear the chest and helping to prevent the disease worsening in lieu of access to other interventions and face-to-face check-ups. The sense of achievement from being able to perform a tune was empowering and left participants feeling ‘high’. Playing the harmonica appeared to be more difficult for those who had learnt over Zoom and perhaps were finding playing, and therefore breathing during the class difficult. However, it is possible that the newcomers to the groups were able to discuss this experience in the present as more meaningful compared to others reflecting on the experience with recall bias. The difficulty in the beginning was a shared experience, but with practice, playing the harmonica becomes habitual and less demanding mentally and physically. From a phenomenological perspective, temporality is part of the lived experience in any phenomenon, and with harmonica playing online, it offered the participants hope, in looking forward to being-with-others again.

### Social need

COVID-19 has meant that many of our social lives have been reliant on online communication with others. Change in lifestyles was particularly amplified for individuals with COPD who were part of a clinically vulnerable group who were told to shield from others to reduce their risk of contracting COVID-19.^31^ The impact of change in social life because of COVID-19 has previously been reported.^31,32^ Singing for Lung Health group participation has been shown to be successfully adapted to online delivery with positive health outcomes.^33^ However, it is not clear to what extent clinical improvements are due to the social nature of music therapy or the art of music making itself. Further trials are needed to compare face-to-face with remote delivery of arts-in-health groups.

The social lifeline that the Zoom class is offering may continue for some time. With COVID-19 still present, many individuals with respiratory disease may be wary of returning to groups. Indeed, the majority of individuals with respiratory disease are keen to continue social distancing and mask wearing,^34^ both of which make playing harmonica in a group difficult. There has been a reported 50% reduction in COPD admissions during COVID-19.^35^ The study authors state that this is likely associated with a reduction in viral infections, because of social distancing measures, which trigger exacerbations and lead to hospitalisations. However, the authors also acknowledge that fear and anxiety may have also contributed to the reduction in admissions.

Providing Zoom-based options for group-based interventions may not only provide the social lifeline for those with access to such technology, as described above, but also reduce infection risk.

### An adjunct

The resistance of the harmonica at the mouth means that increased pressures are likely needed to be generated on the inhalation and exhalation to trigger and maintain flow for the breath. From a rehabilitation perspective, the harmonica appears to act as an inspiratory muscle trainer on the inspiratory drawing action; and as a PEP device when blowing into the harmonica. Inspiratory muscle training has been shown to be effective to improve respiratory muscle strength and reduce dyspnoea in individuals with COPD.^36^ Furthermore the use of PEP devices in COPD improves health outcomes such as symptom reduction and improvements in exercise capacity,^37^ respiratory pressures^38^ and is recommended in national guidelines.^39^ Playing the harmonica may have similar effects to performing pursed-lip breathing which is also a recommended technique to help improve breathlessness for individuals with COPD.^40^ Participants repeatedly commented that drawing was harder than blowing into the harmonica and that with blowing there was a sense of release or ease, particularly by those with more experience. Further mechanistic studies are warranted to investigate the potential of the harmonica as a muscle trainer an airway clearance adjunct.

### Strengths and limitations

This study provides in-depth experiences of individuals who are both very experienced and relatively novice at playing the harmonica. This enabled rich and varied accounts of the lived experiences of harmonica playing. However, we acknowledge that experiences of participants who have previously participated in harmonica groups, but subsequently dropped out, were not gained. Therefore, discussions here may be more biased to positive experiences. Nevertheless, the open-ended nature of questioning in combination with the fact that AL was previously unknown to the participants enabled individuals to be forthright in verbalising not only perceived benefits but also detailing difficulties experienced. Further limitations exist due to the research being performed during the COVID-19 pandemic. New members to the group only had experience of online group participation without comparison to face-to-face sessions.

The sample of participants was unique. Many had combined previous experiences of both participating in PR and SLH groups. This could limit the transferability of the findings to those individuals who have not previously participated in group-based interventions. However, these combined experiences enabled a greater reflection of the meaning of harmonica playing in context with other interventions which is valuable for clinicians and patients to understand. The themes of ‘Gives you a high’ and ‘Needing the Zoom class’ associated with the ‘buzz’ social activities give, are likely effects independent of the group (healthy or not) and the type of activity, whether that be harmonica or any other group-based activity. However, these themes may reduce the above-mentioned potential selection bias, as individuals who enjoy the social aspect are likely to continue to play the harmonica, even without having experienced any positive respiratory effects. Further feasibility studies and then adequately powered RCTs investigating social and clinical outcomes of face-to-face and online harmonica groups for individuals with CRD are now warranted.

## Conclusion

Playing the harmonica is a novel intervention for individuals living with CRD. This study investigated the lived experiences of individuals playing the harmonica in a group of others living with CRD. Participants experienced breathing differently because of playing the harmonica. Breathing with the harmonica, particularly during inspiration, was hard at first, but with experience, playing the harmonica offered control of their breathing, in a fun, social activity which made them feel they were preventing their disease from worsening. Further mechanistic and RCTs are warranted to determine the clinical value of harmonica playing when living with COPD.

## Supplemental Material

sj-pdf-1-crd-10.1177_14799731221083315 – Supplemental Material for Playing the harmonica with chronic obstructive pulmonary disease. A qualitative study Click here for additional data file. Supplemental Material, sj-pdf-1-crd-10.1177_14799731221083315 for Playing the harmonica with chronic obstructive pulmonary disease. A qualitative study by Jasara N. Hogan, Alexis M. Garcia, Rachel L. Tomko, Lindsay M. Squeglia, and Julianne C. Flanagan in Journal of Interpersonal Violence
